# Tolerance and antioxidant response to heavy metals are differentially activated in *Trichoderma asperellum* and *Trichoderma longibrachiatum*

**DOI:** 10.7717/peerj.19016

**Published:** 2025-02-24

**Authors:** Diego Helman Zapata-Sarmiento, Aida Araceli Rodríguez-Hernández, Gabriela Sepúlveda-Jiménez, Mario Rodríguez-Monroy

**Affiliations:** 1 Departamento de Biotecnologia, Instituto Politécnico Nacional Centro de Desarrollo de Productos Bióticos, Yautepec, Morelos, Mexico; 2 Centro de Desarrollo de Productos Bióticos, CONAHCyT-Instituto Politécnico Nacional, Yautepec, Morelos, Mexico

**Keywords:** Lipid peroxidation, Catalases, Peroxidases, *CYS* gene, *GPX* gene, *CAT* gene

## Abstract

Heavy metal pollution reduces the community of soil microorganisms, including fungi from the genus *Trichoderma*, which are plant growth promotors and biological control agents. Because of potential effects on crop productivity, the toxic effects of heavy metals (HMs) in *Trichoderma* are of interest. However, there have been few studies on the biochemical and molecular response to oxidation caused by exposure to copper (Cu), chromium (Cr), and lead (Pb) and whether this antioxidant response is species-specific. In this study, we compared the tolerance of *Trichoderma asperellum* and *Trichoderma longibrachiatum* to Cu, Pb, and Cr and evaluated the expression of genes related to the antioxidant response, including glutathione peroxidase (*GPX*), catalase (*CAT*), and cysteine synthase (*CYS*) as well as the activity of peroxidase and catalase. The isolates of *Trichoderma* were selected because we previously reported them as promotors of plant growth and agents of biological control. Our results revealed that, with exposure to the three HMs, the *Trichoderma* cultures formed aggregates and the culture color changed according to the metal and the *Trichoderma* species. The tolerance index (TI) indicated that the two *Trichoderma* species were tolerant of HMs (Cu > Cr > Pb). However, the TI and conidia production revealed that *T. longibrachiatum* was more tolerant of HMs than *T. asperellum*. The three HMs caused oxidative damage in both *Trichoderma* species, but the enzyme activity and gene expression were differentially regulated based on exposure time (72 and 144 h) to the HMs and *Trichoderma* species. The main changes occurred in *T. asperellum*; the maximum expression of the *GPX* gene occurred at 144 h in response to all three HMs, whereas the *CAT* gene was upregulated at 72 h in response to Cu but downregulated at 144 h in response to all three HMs. The *CYS* gene was upregulated in response to the three metals. The peroxidase activity increased with all three HMs, but the catalase activity increased with Cu and Pb at 72 h and decreased at 144 h with Pb and Cr. In *T. longibrachiatum*, the *GPX* gene was upregulated with all three HMs at 72 h, the *CAT* gene was upregulated only with Pb at 72 h and was downregulated at 144 h with HMs. Cr and Cu upregulated *CYS* gene expression, but expression did not change with Pb. The peroxidase activity increased with Cu at 144 h and with Cr at 72 h, whereas Pb decreased the enzyme activity. In contrast, catalase activity increased with the three metals at 144 h. In conclusion, *T. longibrachiatum* was more tolerant of Cu, Cr, and Pb than was *T. asperellum*, but exposure to all three HMs caused oxidative damage to both *Trichoderma* species. Peroxidases and catalases were activated, and the expression of the genes *GPX* and *CYS* was upregulated, whereas the *CAT* gene was downregulated. These findings indicate that the antioxidant response to HMs was genetically modulated in each *Trichoderma* species.

## Introduction

Heavy metals (HMs) are defined as chemical elements with an atomic number greater than 20, a density greater than five g cm^−3^, and an origin from a natural source. According to these criteria, cadmium (Cd), lead (Pb), mercury (Hg), chromium (Cr), nickel (Ni), copper (Cu), and zinc (Zn) are HMs (Ali & Khan, 2018). The natural sources of HMs are minerals, volcanic eruptions, and rock fragments (Zhang & Wang, 2020), but other principal sources of HMs are anthropogenic activities as mining, the textile industry, paint manufacturing, wastewater irrigation, and the intensive use of agrochemicals such as pesticides and fertilizers (Alengebawy et al., 2021). In cultivated soils, the excessive use of Cu- and Pb-based pesticides is one of the main factors that increases the concentrations of these metals in soils (Facchinelli, Sacchi & Mallen, 2001). Similarly, the intensive use of fertilizers for long periods increases the accumulation of Cu, Cd, and Zn in agricultural soils, which reduces soil fertility and crop productivity (Alengebawy et al., 2021). HMs also reduce the soil microbial community (bacteria and fungi) and activity of microbial enzymes, which are indicators of soil quality and health (Keiblinger et al., 2018; Raiesi & Sadeghi, 2019); therefore, HMs are toxic to plants and the soil microorganisms. In fungi, Cu and Cr toxicity is due to the production of reactive oxygen species (ROS) that damage cells (Belozerskaya & Gessler, 2007; Viti et al., 2014). Pb does not directly participate in the reactions that lead to ROS production, but the metal accelerates the oxidation of DNA, proteins, and antioxidant enzymes that counteract oxidative damage (Gurer & Ercal, 2000).

The oxidative damage to soil fungi caused by HMs is ecologically relevant to crop productivity because there are fungi that are plant growth promoters and agents of biological control of pathogens (Saeed et al., 2021). In particular, fungi of the genus *Trichoderma* carry out these functions in their interaction with plants of agricultural importance; in addition, *Trichoderma* fungi induce defense responses in plants under abiotic and biotic stress (Macías-Rodríguez et al., 2020; Poveda, 2022). However, there have been few studies on HM tolerance that have focused on the oxidative damage and antioxidant response of *Trichoderma* species that are plant growth promoters and agents of biological control of pathogens. Generally, previous studies have evaluated tolerance to HMs in *Trichoderma* species isolated from soils and water contaminated by HMs (Tansengco et al., 2018; Liaquat et al., 2020; Sun et al., 2020; Mushtaq, Bareen & Tayyeb, 2023). Other studies have shown that Cr causes oxidative damage in *Trichoderma lixii* isolated from electroplating wastewater (Kumar & Dwivedi, 2019), and that Cu-based fungicides also cause oxidative damage in *Trichoderma asperellum* isolated from onion crops (Pérez-Torres et al., 2020). But the results on antioxidant enzyme activity are contradictory and suggest that this antioxidant response may depend on the particular *Trichoderma* species and the specific HM. We previously reported that the activity of catalases and peroxidases in three isolates of *T. asperellum* increased with exposure to Cu-based fungicides, and that the enzyme activity depended on the specific isolate of *T. asperellum* (Pérez-Torres et al., 2020). In contrast, catalase activity in *T. lixii* decreased with Cr, and peroxidase and superoxide dismutase activities depended on Cr concentration (Kumar & Dwivedi, 2019). In addition, studies on the expression of detoxification-related genes in response to the exposure of *Trichoderma* to HM are scarce. The transcriptomic analysis of *Trichoderma harzianum* treated with Cd showed that the gene expression of proteins with oxidoreductase activity increased, suggesting that these proteins counteract oxidative damage and other toxic effects of Cd (Oshiquiri et al., 2020). Based on these findings, we evaluated tolerance; oxidative damage; expression of detoxification-related genes such as glutathione peroxidase (*GPX*), catalase (*CAT*), and cysteine synthase (*CYS*); and catalase and peroxidase activity in *T. asperellum* and *Trichoderma longibrachiatum* exposed to Cu, Cr, and Pb. These *Trichoderma* species were selected because we previously evaluated their potential as promotors of plant growth, agents of biological control, and inducers of plant resistance (Ortega-García et al., 2015; Zapata-Sarmiento et al., 2020; Rodríguez-Hernández et al., 2023; Camacho-Luna et al., 2021; Camacho-Luna et al., 2023). Cu and Cr were evaluated because their toxicity is mediated by the production of ROS (Belozerskaya & Gessler, 2007; Viti et al., 2014), whereas Pb is a metal that accelerates the oxidation of DNA and proteins, including those of antioxidant enzymes (Gurer & Ercal, 2000). The accumulation of these HMs in soils is caused by the intensive use of agrochemicals (Alengebawy et al., 2021). Thus, this study contributes to the knowledge of the genetic and biochemical mechanisms related to the antioxidant response to Cu, Cr, and Pb exposure in two *Trichoderma* species with potential agricultural use.

## Materials & Methods

### Evaluation of the growth, conidia production, and tolerance of *Trichoderma asperellum* and *T. longibrachiatum* exposed to Cu, Cr, and Pb

*Trichoderma asperellum* was previously isolated and identified by Ortega-García et al. (2015), and *T. longibrachiatum* was isolated and identified by Camacho-Luna et al. (2022). Fungal strains were grown in a culture medium of potato, dextrose, and agar (PDA, Bioxon™, Becton Dickinson from Mexico) at 25 ± 2 °C with a 12 h light:12 h dark cycle and after 8 d, conidial suspensions (1 × 10^7^ conidia mL^−1^) were obtained. Due to our previous study in which we reported that a Cu concentration of 100 mg L^−1^ is not lethal for *T. asperellum* (Pérez-Torres et al., 2020), we decided to compare the tolerance, oxidative damage, activity of antioxidant enzymes, and genetic expression in the two *Trichoderma* species exposure to Cu, Cr, and Pb at 100 mg L^−1^. The Erlenmeyer flasks contained a culture medium of potato and dextrose (Difco; BD, Franklin Lakes, NJ, USA), and 100 mg L^−1^ of each HM. Cu was added as CuSO_4_ × 5 H_2_O (Sigma-Aldrich Co., St. Louis, MO, USA), Cr was added as K_2_Cr_2_O_7_ (J.T. Baker; Avantor Performance Materials, Radnor, PA, USA), and Pb was added as Pb (NO_3_)_2_ A.C.S. (Fermont, Productos Químicos Monterrey, Monterrey, N.L., Mexico). For each HM and *Trichoderma* species, four Erlenmeyer flasks were prepared and inoculated with one mL of conidial suspension (1 × 10^7^ conidia mL^−1^). The controls were Erlenmeyer flasks inoculated with each *Trichoderma* species without HMs. After 6 d, the mycelia were recovered and dried, and the dry weight (DW) was obtained. To quantify the conidia production, samples (one mL) of culture medium broth were collected and conidia were counted in a Neubauer hemocytometer. Conidia production was expressed as the number of conidia per gram DW. Images of *Trichoderma* cultures developed in Erlenmeyer flasks and the bottom of the culture were obtained using a Samsung camera (64 megapixels, F1.8). The tolerance index (TI) was calculated based on the dry biomass using the equation TI = (Dry biomass with heavy metal/Dry biomass without heavy metal) according to Lobos et al. (2021). A second experiment was performed under the same experimental conditions, and fresh mycelial biomass was collected at 72 and 144 h and used to evaluate lipid peroxidation, gene expression, and peroxidase and catalase activity.

### Determination of malondialdehyde (MDA) content

Lipid peroxidation was determined by measuring thiobarbituric acid reactive substances (TBARS) as described previously by Alia, Prasad & Pardha (1995) and Pérez-Torres et al. (2020). Mycelial fresh (300 mg) was finely ground in a mortar with one mL of trichloroacetic acid 5% (w/v) and centrifuged at 15,000 × g at 4 °C for 10 min. The supernatant was recovered, one mL of thiobarbituric acid (TBA; 0.5% in 20% trichloroacetic acid) was added, and the mixture was incubated at 95 °C in a water bath for 30 min and then centrifuged at 9,500 × g at 15 °C for 10 min. The absorbance of the supernatant was measured at 532 nm in a spectrophotometer (Thermo Spectronic^®^, Genesys Model 2). The absorbance values were used to calculate the MDA content using a standard curve prepared using known concentrations of MDA, and the results were expressed as nmol of MDA g^−1^ fresh weight. The MDA index, which measures the MDA content of mycelium samples of *Trichoderma* treated with and without metal, was calculated.

### RNA isolation and RT−qPCR relative expression analysis

After 72 and 144 h of exposure to Cu, Pb, and Cr, total RNA was extracted from fresh mycelia of *T*. *asperellum* and *T*. *longibrachiatum* with TRIzol^®^ Reagent for small scale isolation (Invitrogen, Carlsbad, CA, USA) and treated with RNase-free DNase I (Thermo Fisher Scientific Carlsbad, CA, USA). Isolated total RNAs were kept at −80 °C until use for complementary DNA (cDNA) synthesis. The total RNA concentration was calculated with an Epoch-2 microplate reader (Biotek^®^ Winooski, VT, USA). The integrity of RNA was checked using 1.2% agarose gel electrophoresis under denaturing conditions. RT–qPCR was performed according to Rodríguez-Hernández et al. (2014) with a StepOne Real-Time PCR Detection System (Applied Biosystems, Waltham, MA, USA), and StepOne Software v2.1 (Applied Biosystems). For the synthesis and quantification of cDNA, the iTaq Universal SYBR^®^ Green One-Step kit (Bio-Rad, Hercules, CA, USA) was used. RT-qPCR was performed in 10 µl reaction mixture containing 100 ng of total RNA as template, five µl of iTaq universal SYBR Green reaction Power mix (2X), 300 nM of each oligonucleotide, and 0.125 µl of iScript reverse transcriptase. All samples were amplified in triplicate as follows: 10 min at 50 °C (cDNA synthesis) and 1 min at 95 °C (polymerase activation) followed by 40 cycles at 95 °C (DNA denaturation) and 1 min at 60 °C (tubulin), 58 °C (*GPX*, gluthatione peroxidase and *CYS*, cysteine synthase), and 55 °C (*CAT*, catalase). The primers corresponding to the *Tubulin* gene were *TUB-F* 5′-GACACTACACTGAGGGTGCT-3′ and *TUB-R* 5′-GTATGACGGGTTGGACAGCT-3′; the primers corresponding to the *Catalase* gene were: *CAT-F* 5′-ACTGCTGAGGGTTGCCCAAT-3′ and *CAT-R* 5′-CGAATTCACCATATGCACCAG-3′, the primers corresponding to the *Gluthatione peroxidase* gene were: *GPX-F* 5′-ATTCAGCGGACAAATTCAGTGC-3′  and *GPX-R* 5′-CAGCCTTACGGAGCTCCG-3′; and the primers corresponding to the *Cysteine synthase* gene were: *CYS-F*  5′-ATGTTCCGACAAACTGCGCAG-3′and *CYS-R* 5′-GCAGCCGGTCTCCTCAG-3′. The melting curve was generated with cycles of 5–95 °C for 15 s, with increases of 0.5 °C at each cycle. We used tubulin as a reference for all experiments, and the gene expression levels were evaluated using the 2^−ΔΔ*CT*^ method (Livak & Schmittgen, 2001). The relative expression data were normalized with the assumption that the control has a value of 1, and with respect to this assumption, the fold change in gene expression level was calculated for each treatment per gene. For each sample, three replicates (*n* = 3) and their respective technical replicates were analyzed.

### Determination of enzyme activities

Peroxidase activity was determined using the methods described by Stasolla & Yeung (2007) and Pérez-Torres et al. (2020). Briefly, fresh mycelia (300 mg) were extracted with sodium phosphate buffer (50 mM, pH 6.0) containing EDTA (100 mM), DTT (one mM), and PMSF (one mM). The extract was centrifuged, and the recovered supernatant was used as the enzyme extract. The protein content was measured by Bradford’s (1976) method and calculated with a standard curve constructed with bovine serum albumin. The reaction enzyme was prepared with the enzyme extract using guaiacol as the substrate. The tetraguaiacol content (extinction coefficient of 26.6 mM cm^−1^) was calculated. The enzyme activity was expressed as the tetraguaiacol content per minute per milligram of protein.

Catalase activity was determined by the methods of Beers & Sizer (1952) and Pérez-Torres et al. (2020). Fresh mycelium (300 mg) was ground with extraction buffer containing sodium phosphate (100 mM at pH 7.0), EDTA (one mM), DTT (one mM), and PMSF (one mM). The extract was centrifuged and used as the enzyme extract. The enzyme reaction was developed using an H_2_O_2_ solution at 30% as substrate. The absorbance at 240 nm was measured and used to calculate the H_2_O_2_ content using an extinction coefficient for H_2_O_2_ of 0.04 mM cm^−1^. The peroxidase activity was expressed as the H_2_O_2_ content per minute per milligram of protein.

### Statistical analysis

The mean and standard deviation (*n* = 4) of the dry biomass, TI, conidia production, MDA index, and enzyme activity data were analyzed using a one-way analysis of variance (ANOVA) and Tukey’s test (*p* < 0.05) to determine significant differences between the treatments. Statistical analysis of normalized expression was performed with a one-way ANOVA using GraphPad Prism version 9.5.0 (GraphPad Software, San Diego, CA, USA). A Dunnett post hoc test was used for multiple comparisons.

## Results

### Changes in growth, conidia production, and tolerance of *T. asperellum* and *T. longibrachiatum* to Cu, Cr, and Pb

**Figure 1:**
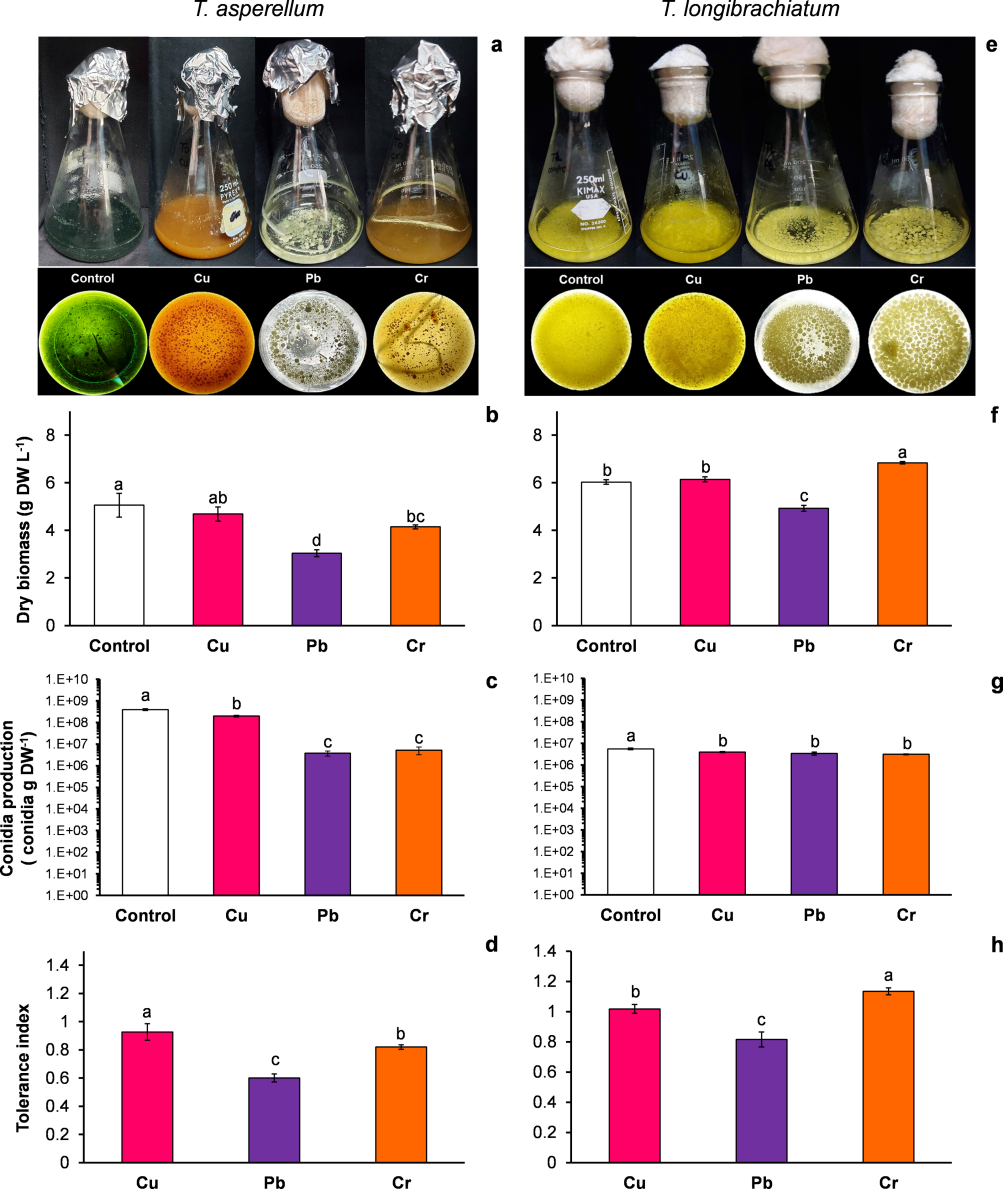
Growth, conidia production and tolerance of *Trichoderma asperellum* and *Trichoderma longibrachiatum* exposure to Cu, Pb, and Cr for 144 h. The images of liquid cultures and culture bottoms in Erlenmeyer flasks of *T. asperellum* (A) and *T. longibrachiatium* (E) show the changes in mycelial growth and culture pigmentation. Dry biomass (B and F), conidia production (C and G), and tolerance index (D and H). The control cultures were grown without metals. The mean ± standard deviation (*n* = 4) was calculated and analyzed using a one-way analysis of variance (ANOVA), and significant differences in dry biomass and conidia production between treatments were determined using Tukey’s test (*P* < 0.050).

*T. asperellum*: Without HM (control), the color of the *T. asperellum* culture was bright green, and the hyphal growth was disaggregated; however, with Cu, the culture color changed to orange, and with Pb and Cr, the intensity of the green color decreased. In the presence of all three metals, the hyphae grew and formed aggregates (Fig. 1A). With exposure to Cu, the dry biomass of *T. asperellum* was similar to that of the control. However, with exposure to Pb and Cr, the dry biomass decreased by 40% and 18%, respectively, compared with that of the control (Fig. 1B). In the presence of Cu, the conidia production of *T. asperellum* decreased but was at the same order of magnitude as that of the control. With exposure to Pb and Cr, conidia production decreased significantly by two orders of magnitude in comparison with that of the control (Fig. 1C). The TI values with exposure to Cu, Pb, and Cr were 0.92, 0.60, and 0.82, respectively (Fig. 1D).

*T. longibrachiatum*: Without exposure to HM (control), the *T. longibrachiatum* culture had a bright yellow color, and hyphal growth was disaggregated. With Cu, the culture color was pale yellow, and with Cr and Pb the culture color was lime green; with exposure to all three metals, the hyphae grew and formed aggregates (Fig. 1E). With exposure to Cu, the dry biomass was similar to that of the control, whereas with exposure to Pb, the dry biomass decreased by 18% compared with that of the control. In contrast, the dry biomass increased with Cr compared with that of the control (Fig. 1F). In the presence of the three metals, the conidia production of *T. longibrachiatum* decreased but was at the same order of magnitude as that of the control (Fig. 1G). The TI values with exposure to Cu, Pb, and Cr were 1.01, 0.81, and 1.33, respectively (Fig. 1H). These TI values were greater than those observed in *T. asperellum*.

### Cu, Cr, and Pb cause lipid peroxidation in both *Trichoderma* species

**Figure 2:**
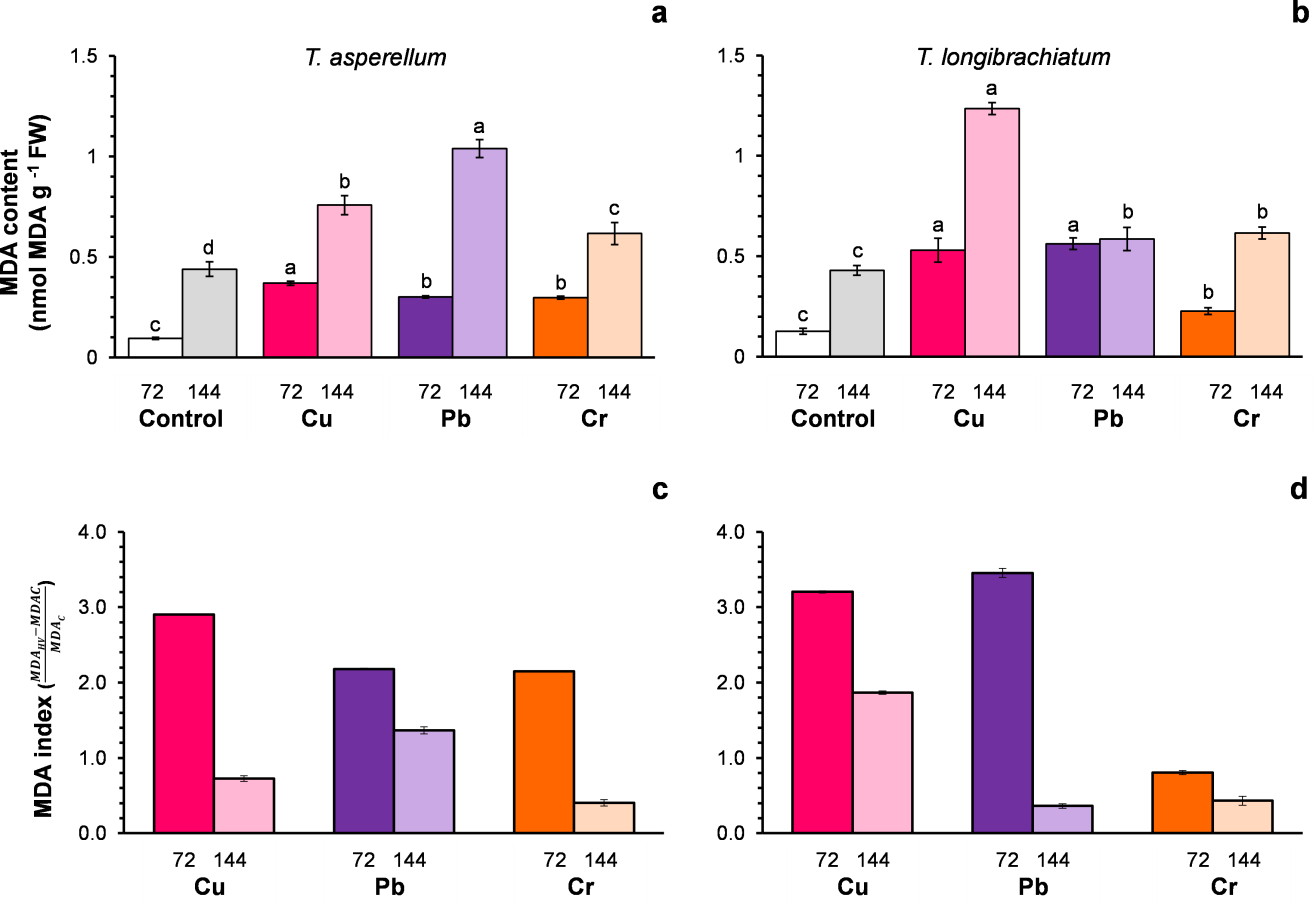
Malondialdehyde (MDA) content (A and B) and the MDA index (C and D) of *Trichoderma asperellum* (A and C) and *Trichoderma longibrachiatum* (B and D) exposure to Cu, Pb, and Cr for 72 and 144 h. The control cultures were grown without metals. The mean ± standard deviation (*n* = 4) was calculated and analyzed using a one-way analysis of variance (ANOVA), and significant differences in the MDA content between treatments were determined using Tukey’s test (*P* < 0.050).

For both *Trichoderma* species without metals (control), the MDA content changed with the incubation time of the culture (Figs. 2A–2B). For this reason, the MDA index was assumed to be an indicator of oxidative stress. In both *Trichoderma* species, the MDA index values were highest at 72 h after exposure to metals. In *T. asperellum*, the increase in the MDA index at 72 h exhibited a rank order of Cu > Pb = Cr, whereas in *T. longibrachiatum,* the increase in the MDA index exhibited a rank order of Pb > Cu > Cr (Figs. 2C–2D).

### Cu, Pb, and Cr cause differential expression of detoxification-related genes in *T. asperellum* and *T. longibrachiatum*

**Figure 3:**
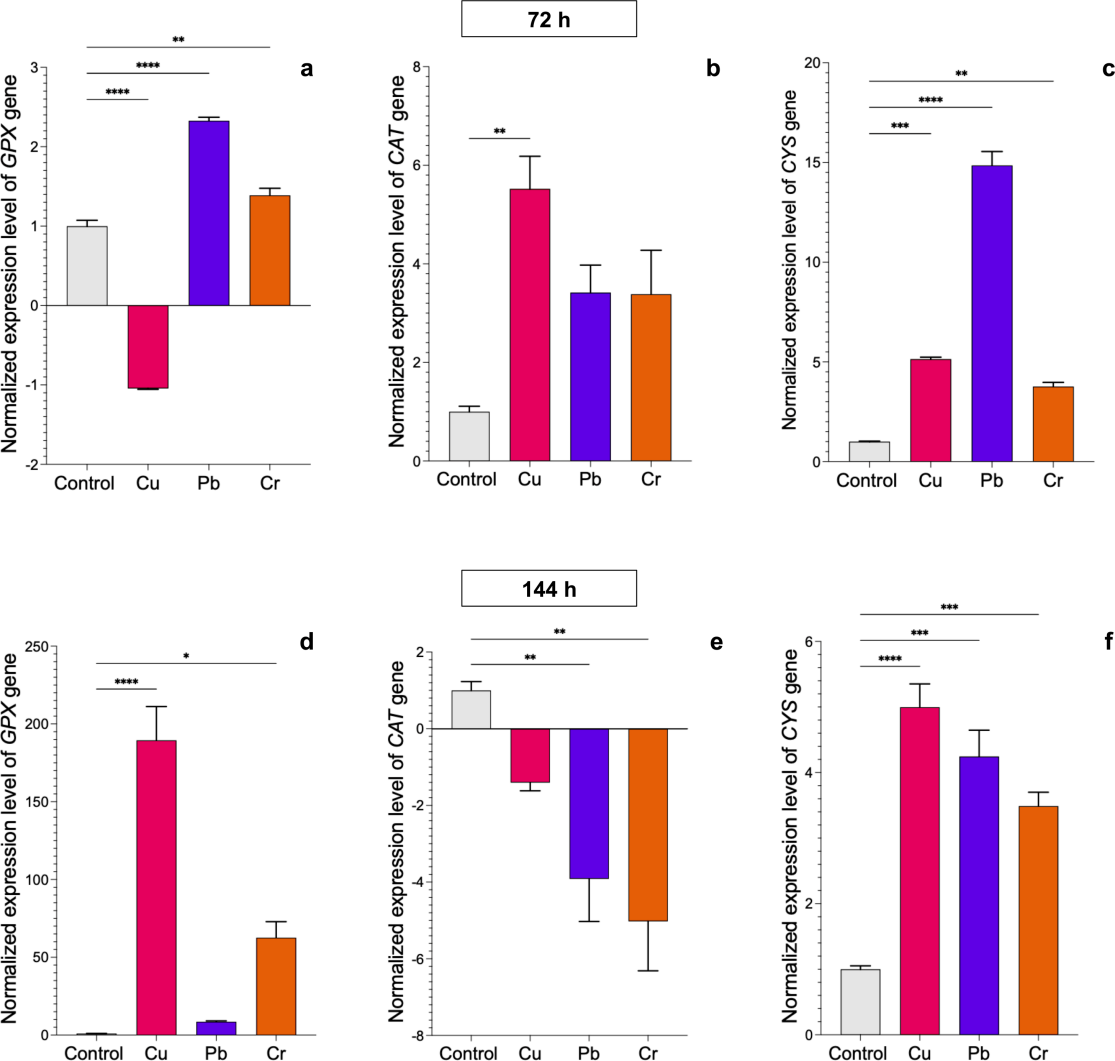
Normalized expression levels of the *CAT*, *GPX*, and *CYS* genes in *Trichoderma asperellum* exposure to Cu, Pb, and Cr for 72 and 144 h. The control cultures were grown without metals. The relative expression of *CAT*, *GPX*, and *CYS* was normalized against that of the *Trichoderma tubulin* (*TUB*) gene. The relative expression data were normalized with the assumption that the control has a value of 1, and with respect to this, the fold change in gene expression level was calculated for each treatment per gene. The experiments were repeated in triplicate. A one-way ANOVA with a Dunnett post hoc test was used for multiple comparisons, and asterisks indicate significant differences with (*) *P* < 0.0332, (**) *P* < 0.0021, (***) *P* < 0.0002 and (****) *P* < 0.0001.

We evaluated the transcriptional profiles of the *GPX*, *CAT*, and *CYS* genes, which encode enzymes involved in the antioxidative response, in both *Trichoderma* strains after exposure to HMs. In *T. asperellum*, expression of the *GPX* gene was upregulated after 72 h of exposure to Pb and Cr, whereas it was downregulated after exposure to Cu (Fig. 3A). The most notable changes with respect to the control were found after 144 h of treatment with the three metals. Expression of the *GPX* gene increased 188.5-fold in response to exposure to Cu, 7.6-fold in response to Pb, and 61-fold in response to Cr (Fig. 3D). Expression of the *CAT* gene was upregulated at 72 h of exposure to Cu (4.6-fold), whereas expression of the gene did not significantly change with exposure to Pb or Cr; however, after 144 h, expression of the gene was downregulated with all three metals (Figs. 3B–3E). Expression of the *CYS* gene was upregulated after 72 h of treatment with all three metals. With Cu, the expression increased up to 4-fold at 72 h and 144 h; with Pb, expression increased 13.8- and 3.2-fold at 72 and 144 h, respectively; and with Cr, expression increased 2.8-fold at 72 h and 2.5-fold at 144 h (Figs. 3C–3F).

**Figure 4:**
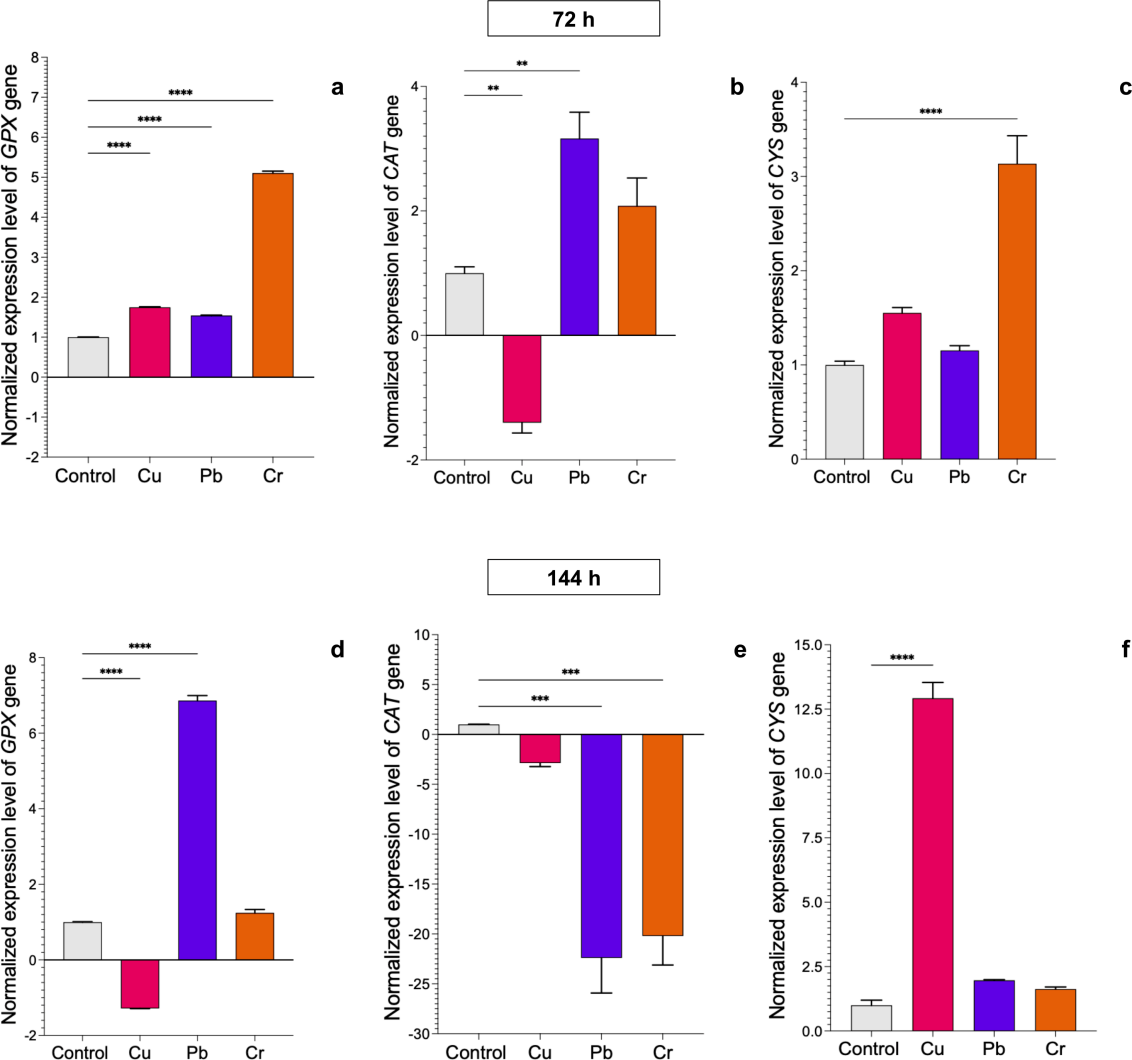
Normalized expression levels of the *CAT*, *GPX*, and *CYS* genes in *Trichoderma longibrachiatum* exposure to Cu, Pb, and Cr for 72 and 144 h. The control cultures were grown without metals. The relative expression of *CAT*, *GPX*, and *CYS* was normalized against that of the *Trichoderma tubulin* (*TUB*) gene. The relative expression data were normalized with the assumption that the control has a value of 1, and with respect to this, the fold change in gene expression level was calculated for each treatment per gene. The experiments were repeated in triplicate. A one-way ANOVA with a Dunnett post hoc test was used for multiple comparisons, and asterisks indicate significant differences with (*) *P* < 0.0332, (**) *P* < 0.0021, (***) *P* < 0.0002 and (****) *P* < 0.0001.

In *T. longibrachiatum*, *GPX* gene expression was upregulated at 72 h of exposure to all three metals: 0.7-fold for Cu, 0.6-fold for Pb, and 4.1-fold for Cr. At 144 h of exposure to Cu, expression was downregulated, and with Pb and Cr exposure, expression was upregulated by 5.9-fold and 0.4-fold, respectively (Figs. 4A–4D). At 72 h, the *CAT* gene was downregulated with Cu and upregulated with Pb (2.2-fold), but at 144 h, the *CAT* gene was downregulated with exposure to all three metals (Figs. 4B–4E). The *CYS* gene was upregulated with exposure to Cr (2.1-fold) at 72 h and with exposure to Cu at 144 h (11.9-fold). Pb did not affect expression of the *CYS* gene (Figs. 4C–4E).

### Cu, Pb, and Cr differentially induce peroxidase and catalase activation in *T. asperellumm* and *T. longibrachiatum*

**Figure 5:**
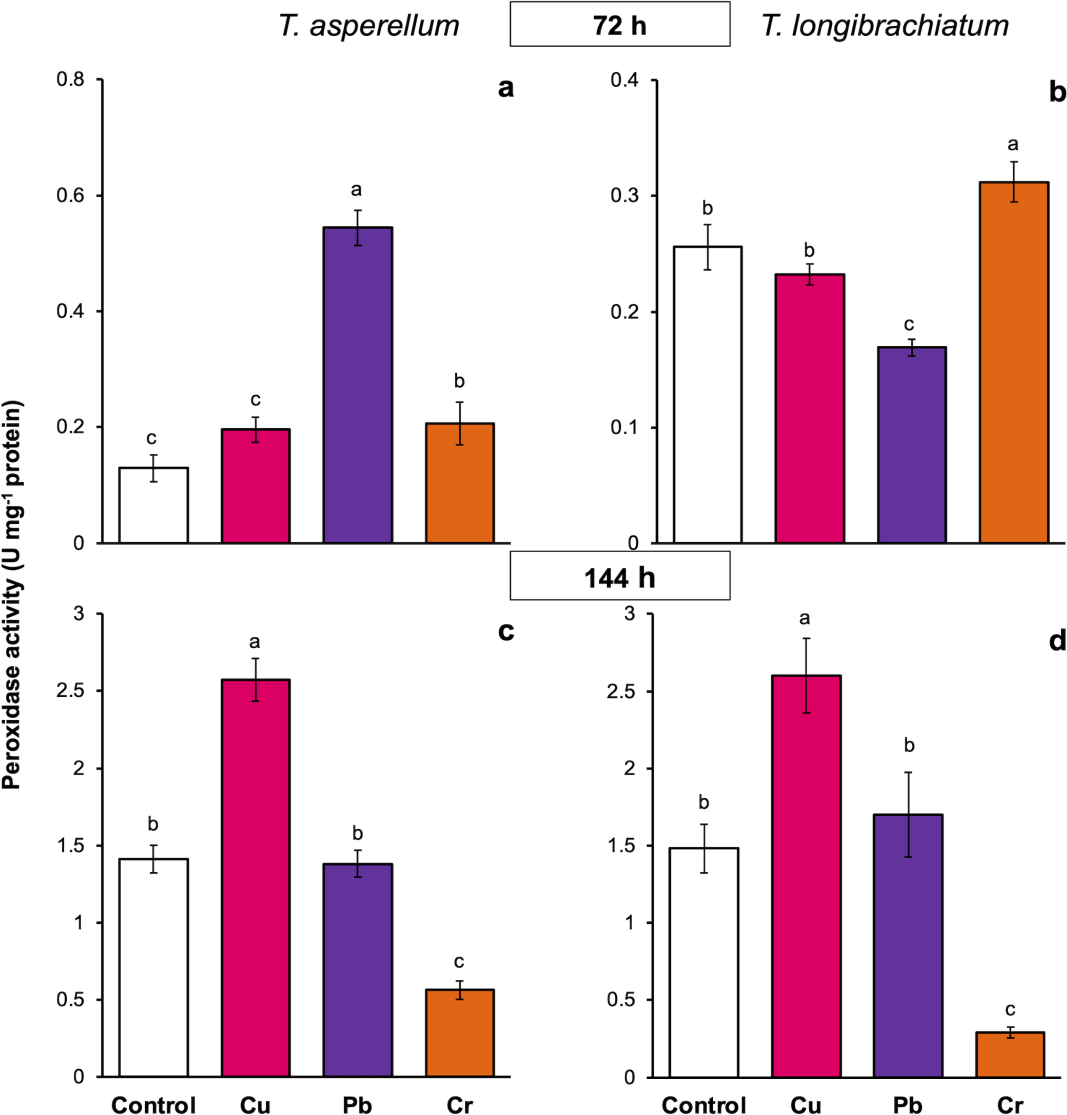
Peroxidase activity in *Trichoderma asperellum* and *Trichoderma longibrachiatum* exposure to Cu, Pb, and Cr for 72 (A and B) and 144 h (C and D). The control cultures were grown without metals. The mean ± standard deviation (*n* = 4) was calculated and analyzed using a one-way analysis of variance (ANOVA). Different lowercase letters indicate significant differences in peroxidase activity between treatments according to Tukey’s test (*P* < 0.05).

The peroxidase activity of both *Trichoderma* species also depended on the exposure time and metal. In both *Trichoderma* species, exposure to Cu for 72 h did not change peroxidase activity, but at 144 h, the enzyme activity increased 0.8-fold compared with that of the controls. Compared with that of the controls, the peroxidase activity of *T. asperellum* increased 3.2-fold after exposure to Pb for 72 h, whereas the enzyme activity decreased in *T. longibrachiatum*. In contrast, in both *Trichoderma* species, exposure to Pb for 144 h did not affect peroxidase activity. Exposure to Cr for 72 h increased peroxidase activity in both *Trichoderma* species, but exposure to Cr for 144 h decreased peroxidase activity in both *Trichoderma* species compared with that in their controls (Fig. 5).

**Figure 6:**
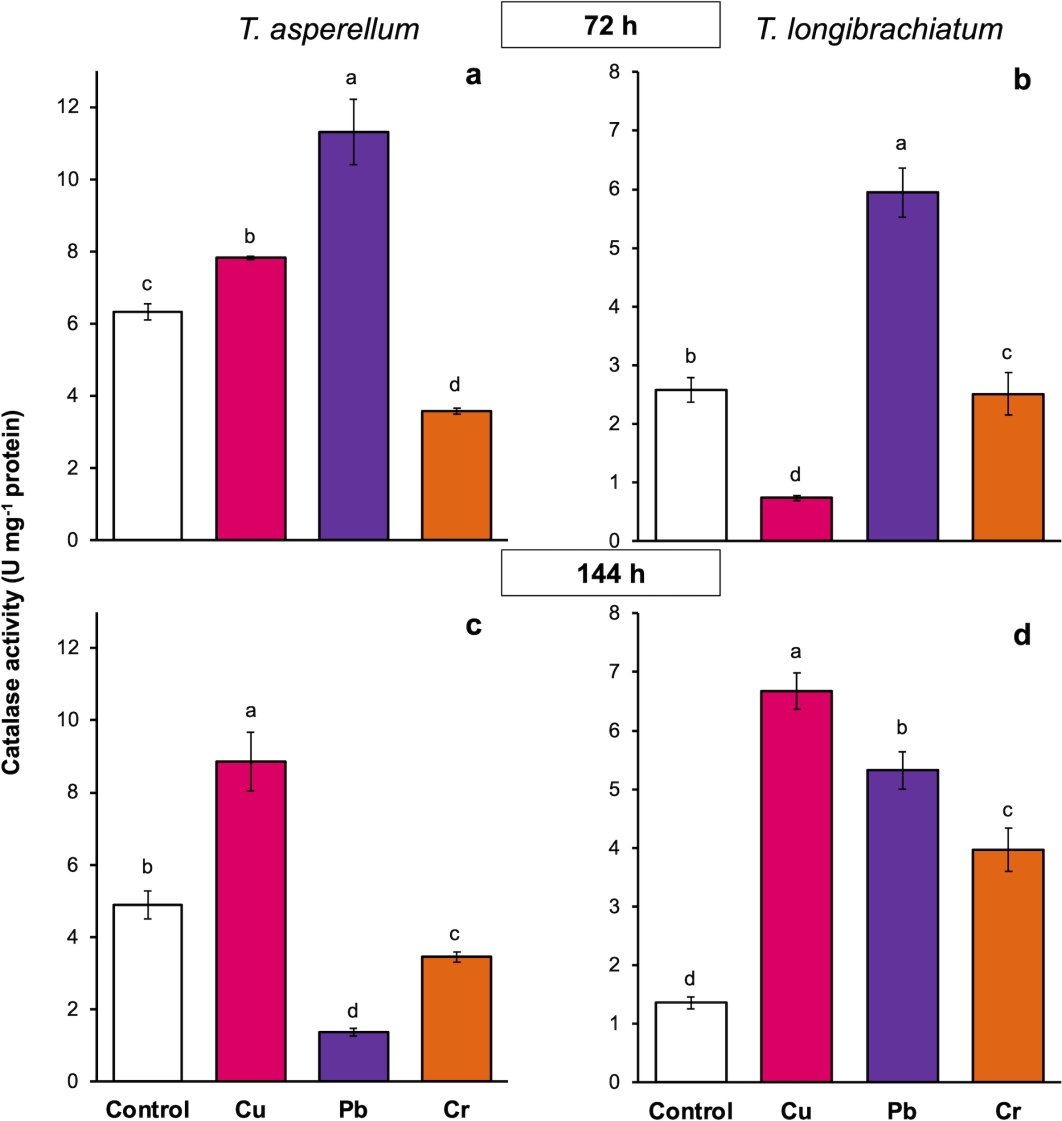
Catalase activity in *Trichoderma asperellum* and *Trichoderma longibrachiatum* exposure to Cu, Pb, and Cr for 72 (A and B) and 144 h (C and D). The control cultures were grown without metals. The mean ± standard deviation (*n* = 4) was calculated and analyzed using a one-way analysis of variance (ANOVA). Different lowercase letters indicate significant differences in catalase activity between treatments according to Tukey’s test (*P* < 0.05).

In *T. asperellum* exposed to Cu for 72 and 144 h, catalase activity increased 0.2- and 0.8-fold, respectively, in comparison with that of the controls. In contrast, in *T. longibrachiatum*, exposure to Cu for 72 h decreased catalase activity, but at 144 h, catalase activity was 3.9-fold greater than that of the control. After exposure to Pb for 72 h, catalase activity increased 0.8- to 1.3-fold in the two *Trichoderma* species; however, after exposure to Pb for 144 h, catalase activity depended on the *Trichoderma* species. In *T. asperellum*, enzyme activity decreased, and in *T. longibrachiatum*, catalase activity increased 2.9-fold compared with that of the controls. In *T. asperellum*, exposure to Cr for 72 and 144 h reduced catalase activity, whereas in *T. longibrachiatum*, exposure to Cr decreased catalase activity at 72 h but increased it by 1.9-fold at 144 h (Fig. 6).

## Discussion

### Mycelial growth, conidia production, and tolerance to Cu, Cr, and Pb depend on the *Trichoderma* species

Exposure of the two *Trichoderma* species to Cu, Cr, and Pb induced changes in mycelium pigmentation and culture morphology. The nature of these changes depended on the *Trichoderma* species and the HM. In general, the mycelium pigmentation and morphology of *T. longibrachiatum* were less affected by exposure to the three HMs compared with those of *T. asperellum*. In addition, the TI values with the three HMs were greater in *T. longibrachiatum* than in *T. asperellum* and conidia production was less affected in *T. longibrachiatum* than in *T. asperellum*; which indicates that *T. longibrachiatum* was more tolerant of Cu, Pb, and Cr than *T. asperellum*. Similarly, it was found that there are differences in tolerance to Cu, Zn, Pb, and Ni in different *Trichoderma* species and strains of the same *Trichoderma* species. Additionally, the species and strains of *Trichoderma* more tolerant to HMs showed fewer changes in color and mycelia physical appearance than species and strains of *Trichoderma* less tolerant to HMs (Siddiquee et al., 2013). With Cu, the color changes were related with the binding capacity of Cu on the fungal cell surface. In *Trichoderma viride* exposed to Cu, the mycelium blue color is due to the binding of Cu to the cellular wall (Anand et al., 2006). Other studies also showed that tolerance to Cu, Pb, and Cr depended on the *Trichoderma* species and HMs: for Cu with *Trichoderma atroviridae*, *Trichoderma harzianum,* and *Trichoderma* spp. (Yazdani et al., 2009; Mohammadian et al., 2017; Maldaner et al., 2021); for Pb with *T. harzianum*, *Trichoderma virens,* and *T. viride* (Liaquat et al., 2020; Tansengco et al., 2018; Joshi et al., 2011); and for Cr with *T. harzianum* and *Trichoderma gamsii* (Liaquat et al., 2020; Tansengco et al., 2018).

### Exposure to HMs causes lipid peroxidation in both *Trichoderma* species

In both *Trichoderma* species without exposure to HMs (controls), the MDA content increased with culture time, suggesting that ROS is an event related to the mycelial growth of *Trichoderma*. In fungi, ROS are produced *via* the enzyme NADPH oxidase (*Nox*), which consists of three catalytic subunits, *NoxA*, *NoxB*, and *NoxC*, and the regulatory subunit *NoxR* (Zhang et al., 2020; Zhang et al., 2021), and the *NoxR* and *Nox1* genes participate in the development and differentiation of *T. atroviride* (Cruz- Magalhães et al., 2019).

Because the MDA content increased with culture time (72 and 144 h), the MDA index was calculated. The greatest increase in the MDA index occurred at 72 h in both *Trichoderma* species exposed to the three HMs, which indicates that the principal process of lipid oxidation or oxidative stress occurred at 72 h, but followed up at 144 h. Similarly, in *Trichoderma* and other fungi, the increase in MDA content caused by exposure to HMs is an indicator of oxidative stress; this was reported for *T. harzianum* with exposure to Cu (Tavsan & Ayar Kayali, 2013), for *T. lixii* with exposure to Cr (Kumar & Dwivedi, 2019), for *Pleurotus ostreatus* and *Pleurotus opuntia* with exposure to Pb and Cr (Zhang et al., 2016; Li et al., 2017; Yadav et al., 2023), and for *Phanerochaete chrysosporium* with exposure to Pb (Huang et al., 2017).

### Cu, Pb, and Cr induced differential expression of detoxification-related genes in *T. asperellum* and *T. longibrachiatum*

Analysis of the expression of the *GPX*, *CAT*, and *CYS* genes in *T. asperellum* and *T. longibrachiatum* under exposure to Cu, Pb, and Cr revealed differential behavior in the two *Trichoderma* species based on metal and exposure time, which indicates that gene activation related to the antioxidant response to the oxidative damage caused by the HMs could indicate a genetic mechanism that is differentially regulated in the two species. The *GPX* gene was differentially expressed between the two *Trichoderma* species. Studies on gene expression related to the antioxidant response in *Trichoderma* are scarce. However, our data were consistent with the results obtained from a transcriptome analysis of *T. harzianum* exposed to Cd, which revealed upregulated expression of proteins with oxidoreductase activity, such as glutathione S transferases (Oshiquiri et al., 2020), suggesting that the *GPX* gene participates in the antioxidant response to HM exposure in *Trichoderma*.

With respect to the expression profile of the *CAT* gene, our results revealed early expression under exposure to Cu in *T. asperellum* and under exposure to Pb in *T. longibrachiatum*. At 144 h, the *CAT* gene was downregulated in both *Trichoderma* species following exposure to HMs. The expression profile of the *CAT* gene in fungi has been poorly studied and specifically in *Trichoderma* fungi, there have been no reports. In the fungi *Morchella spongiola* (Hongyan et al., 2021), *Aspergillus allahabdii* (Cui et al., 2025), and *Volvariella volvacea* (Gao et al., 2020) the *CAT* genes were upregulated with exposure to Cd. In other organisms, the *CAT* genes also participated in the antioxidant response to HM exposure. In tomato plants treated with Cd and Pb, the abundance of *CAT* gene transcripts increased (Aydin et al., 2016). Therefore, our data suggest that the *CAT* gene could be involved in the early response to the oxidative damage caused by exposure to HMs in *Trichoderma*.

The amino acid cysteine is the precursor to metallothioneins, glutathione, and phytochelatins (Clemens, 2006). To determine whether the cysteine synthase (*CYS*) gene is involved in the antioxidant response of both *Trichoderma* species after exposure to Cu, Pb, and Cr, we evaluated its expression level. Notably, in *T. asperellum,* the *CYS* gene was upregulated after 72 h of exposure to all three HMs, whereas in *T. longibrachiatum*, the *CYS* gene was upregulated only after exposure to Cu and Cr but not Pb, which suggests that the *CYS* gene is not involved in the response to Pb. Similarly, expression of the *OASTL* gene that encodes an enzyme involved in cysteine synthesis in *T. harzianum* was induced with exposure to Cd, Pb, Hg, and Zn (Raspanti et al., 2009). Therefore, we suggest that the *CYS* gene participates in the antioxidant response in *Trichoderma*. This is the first report of *CYS* gene expression in the genus *Trichoderma*, specifically in *T. asperellum* and *T. longibrachiatum,* under exposure to Cu, Pb, and Cr. Based on these results, it will be interesting to determine whether the expression of *GPX*, *CAT,* and *CYS* genes is also modulated genetically in different strains of the same *Trichoderma* species.

### Cu, Pb, and Cr caused different peroxidase and catalase activities in *T. asperellum* and *T. longibrachiatum*

Antioxidant enzymes are a cellular mechanism that reduces the toxic effects of excess ROS and is essential for maintaining the cellular redox balance (Belozerskaya & Gessler, 2007). In *Trichoderma*, the antioxidant enzyme activity of some *Trichoderma* species exposed to HMs has been evaluated. However, the results are contradictory and suggest that the antioxidant response may depend on the particular *Trichoderma* species, *Trichoderma* isolate, and HMs. In three isolates of *T. asperellum* exposed to Cu-based fungicides, the activity of catalases and peroxidases depended on the isolate of *T. asperellum* (Pérez-Torres et al., 2020). In contrast, in *T. lixii*, catalase activity has been reported to decrease with Cr, and peroxidase and superoxide dismutase activities depended on the Cr concentration (Kumar & Dwivedi, 2019). On the basis of these results, this study evaluated the peroxidase and catalase activities of two *Trichoderma* species exposed to Cu, Pb, and Cr. Our results revealed that, in both *Trichoderma* species exposed to Cu, the activity of peroxidases and catalases increased principally at 144 h. This result confirms that catalases and peroxidases respond to the oxidative stress induced by Cu-based fungicides in three isolates of *T. asperellum* (Pérez-Torres et al., 2020). In other fungi, such as *Aspergillus niger* (Cavalcanti Luna et al., 2015), *Pleurotus* sp. (Mohamadhasani & Rahimi, 2022), and *Alternaria alternata* (Shoaib, Akhtar & Akhtar, 2015), the activity of catalase or peroxidase also increased with exposure to Cu, suggesting that the increase in antioxidant enzyme activity is to reduce the toxic effects of Cu *via* the degradation of hydrogen peroxide to water.

Our results also revealed that in both *Trichoderma* species exposed to Pb and Cr, peroxidase and catalase activities were dependent on the exposure time and HM. There have been few studies on antioxidant enzyme activity in response to the oxidative stress caused by Pb and Cr in *Trichoderma*. In *T. lixii* exposed to Cr, catalase activity decreased with increasing Cr concentration, whereas peroxidase activity depended on Cr concentration (Kumar & Dwivedi, 2019). In *Phanerochaete chrysosporium*, changes in catalase and peroxidase activities depended on the exposure time to Cd and Pb, and it has been suggested that changes in enzyme activity are a cellular response to protect against the oxidative damage caused by ROS (Zhang et al., 2015; Huang et al., 2017). Thus, our results show that the catalase and peroxidase activities in response to oxidative stress induced by HMs depends on the HM and exposure time to the metal. The antioxidant response to HMs is genetically modulated in each *Trichoderma* species.

### Conclusion

The isolate of *T. longibrachiatum* was more tolerant to Cu, Cr, and Pb than the isolate of *T. asperellum*. However, the three HMs induced oxidative damage in both *Trichoderma* species, and in response to oxidative damage, the activities of peroxidase and catalase and the expression of detoxification-related genes (*GPX*, *CAT*, and *CYS*) were induced differentially in *T. asperellum* and *T. longibrachiatum* depending on the exposure time and HM (Cu, Pb, or Cr). These findings indicate that the antioxidant response to HMs was genetically modulated in each *Trichoderma* species. To our knowledge, this is the first report of *CYS* gene expression in the genus *Trichoderma* and its role in the antioxidant response to Cu, Pb, and Cr. This study contributes to the understanding of the genetic and biochemical mechanisms of the antioxidant response of *Trichoderma* fungi, which have potential use in food crops.

## Supplemental Information

10.7717/peerj.19016/supp-1 Supplemental Information 1 Raw data

10.7717/peerj.19016/supp-2 Supplemental Information 2 Additional data for oligonucleotide design for RT-qPCR

10.7717/peerj.19016/supp-3 Supplemental Information 3 MIQE checklist

## Additional Information and Declarations

### Data Availability

The following information was supplied regarding data availability: The raw measurements are available in the Supplementary File.
