# Supplementary material for: Tolerance and antioxidant response to heavy metals are differentially activated in Trichoderma asperellum and Trichoderma longibrachiatum
Source: PeerJ. 2025 Feb 24;13:e19016. doi: 10.7717/peerj.19016 (PMC11867043; doi:10.7717/peerj.19016)
Supplement: Supplemental Information 2 [file peerj-13-19016-s002.docx]

**Supplementary Table S1.** Additional data for oligonucleotide design for RT-qPCR.

| **Gene** | **Amplicon lenght** | **Indentity (%)** | **Accession number** |
| --- | --- | --- | --- |
| β Tubulina | 118 pb | 100 | AY310326 |
| *GPX* | 115 pb | 100 | XM_024907927 |
| *CYS* | 171 pb | 100 | XM_024903128 |
| *CAT* | 181 pb | 100 | XM_024905174 |
